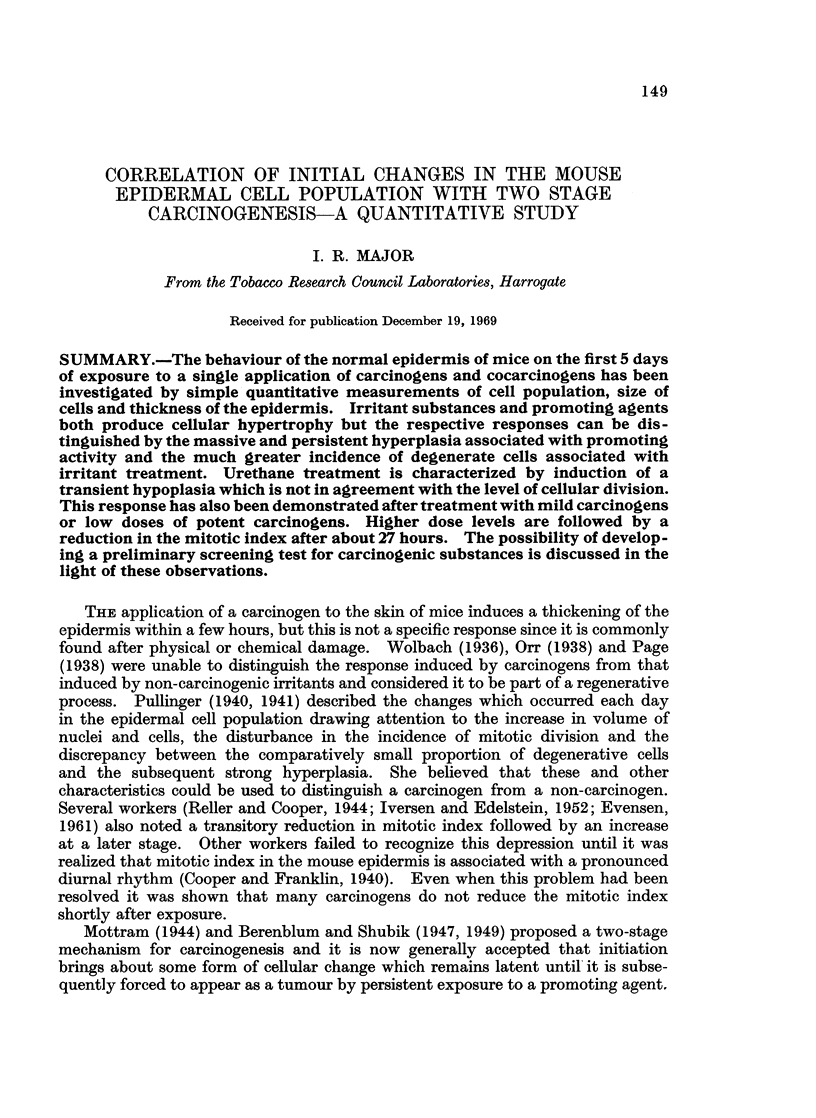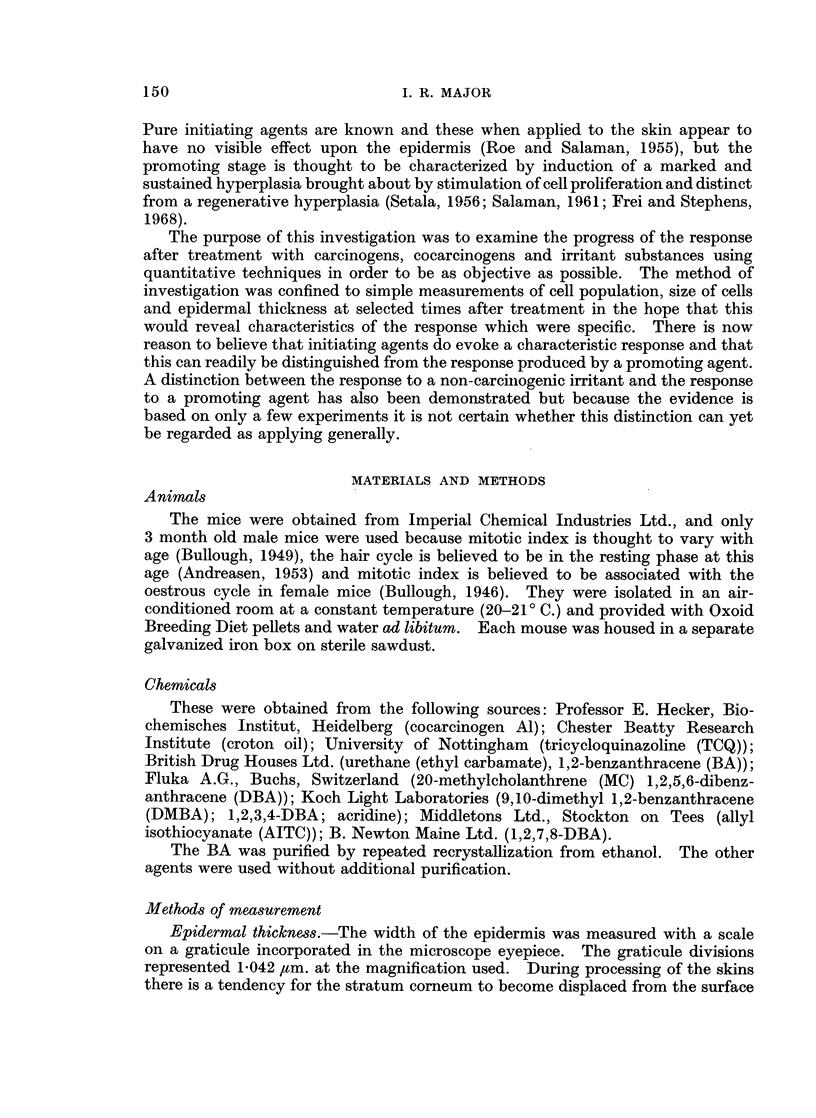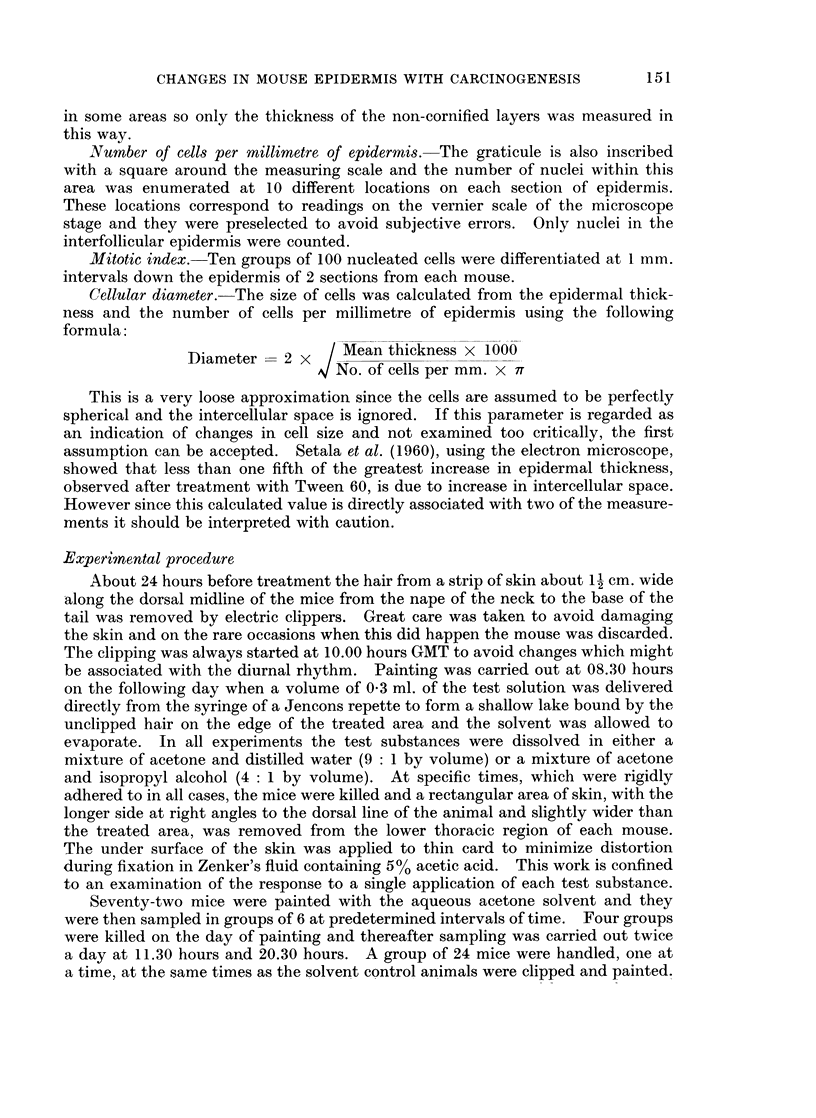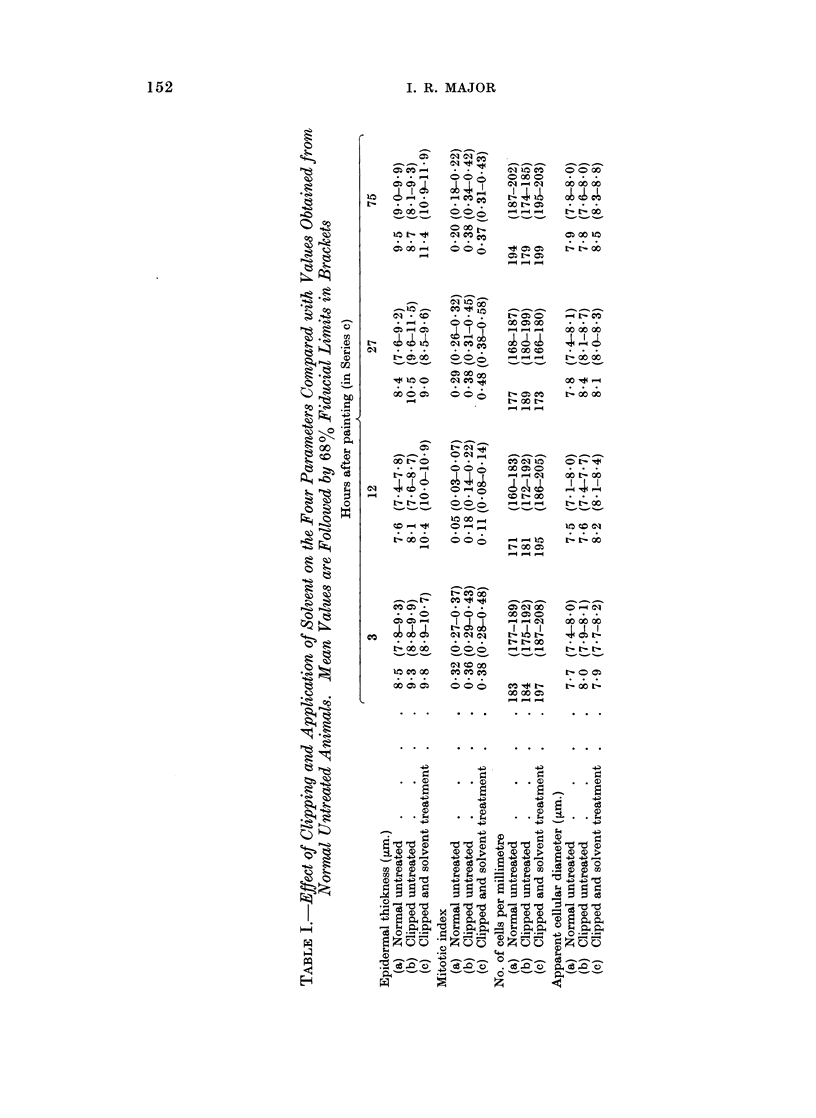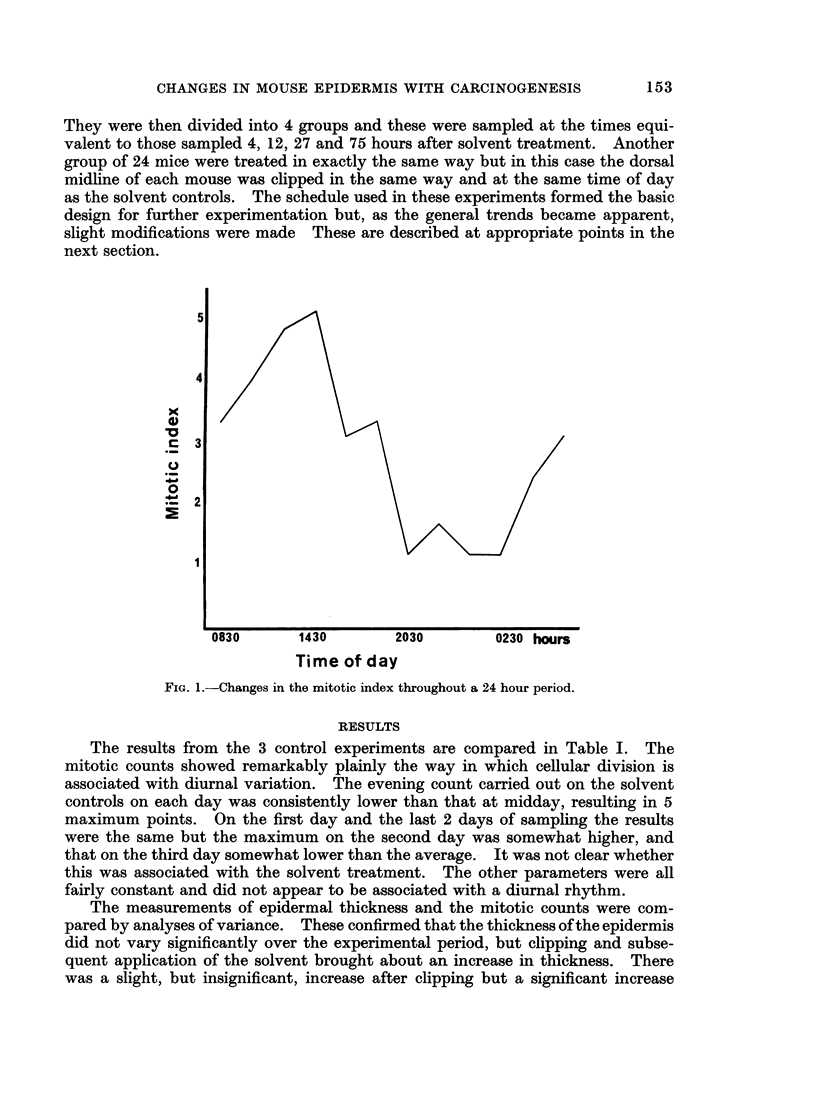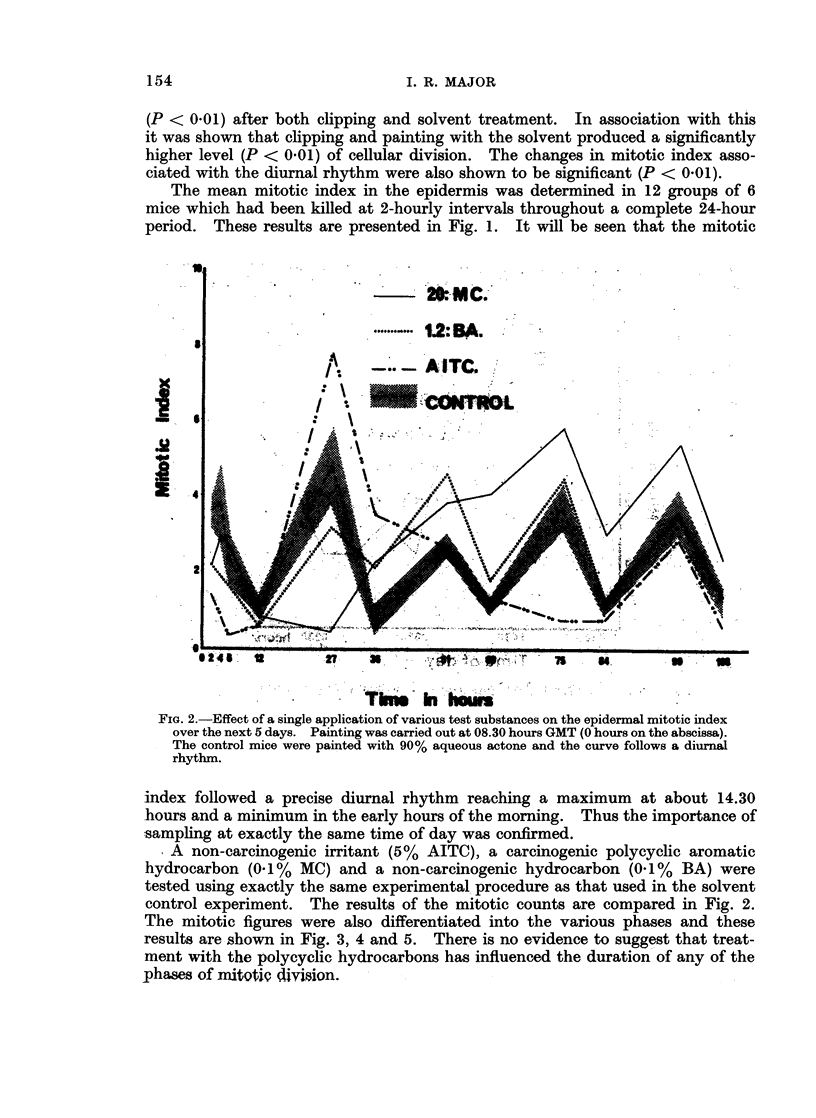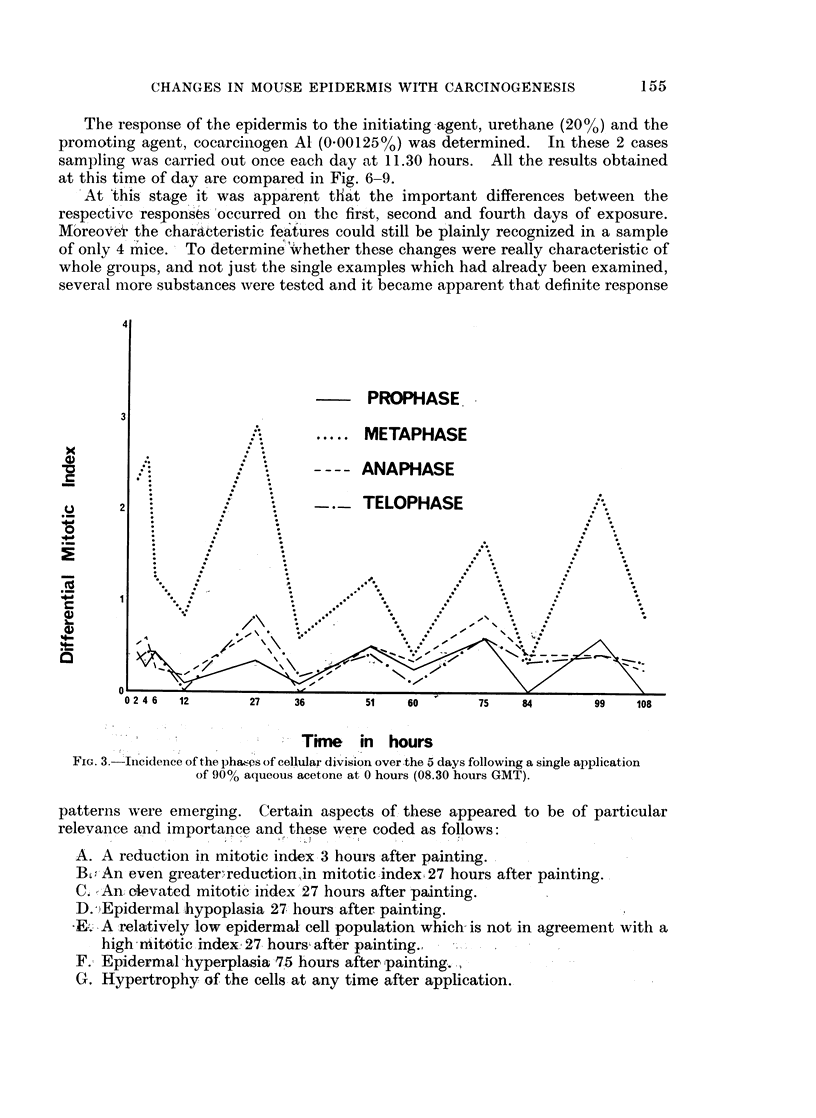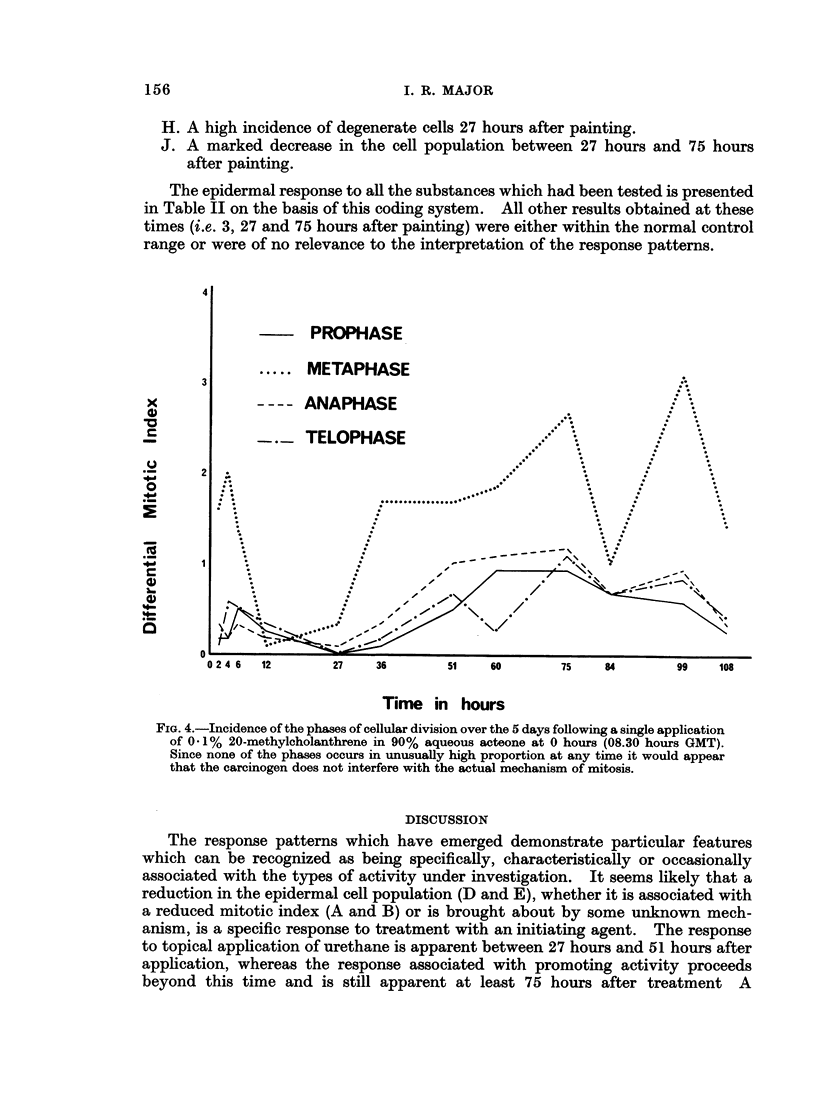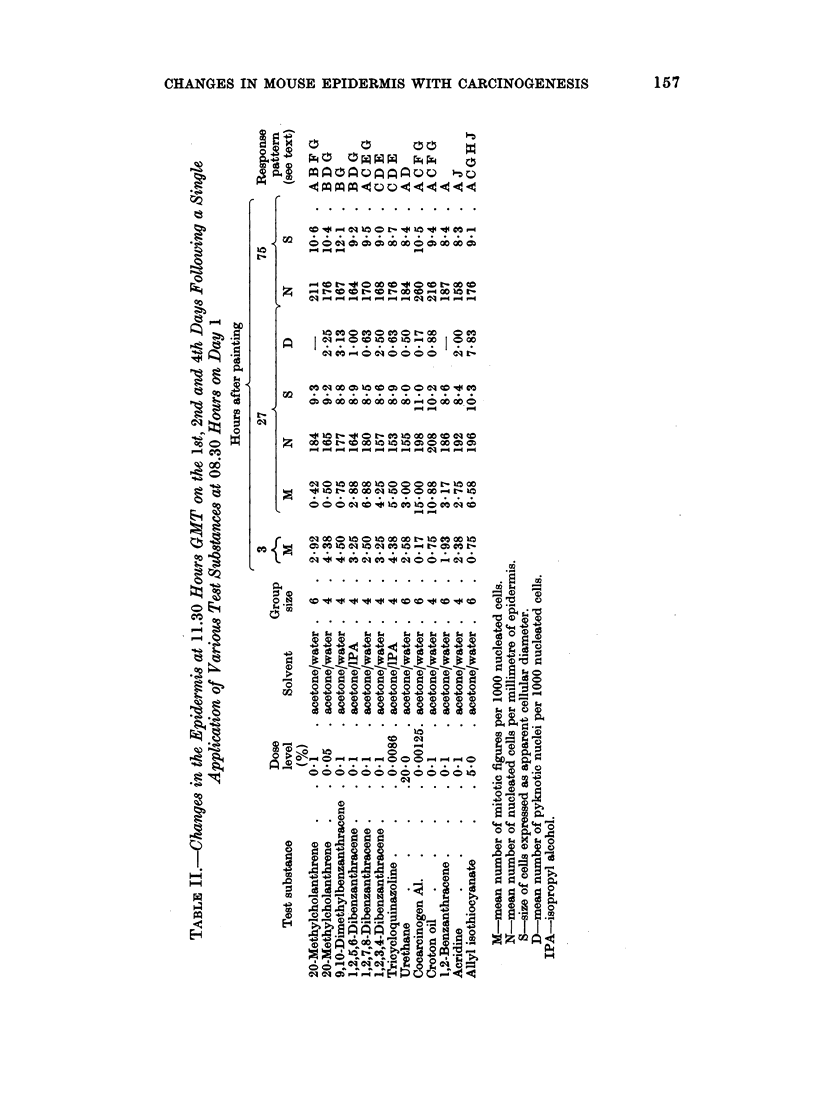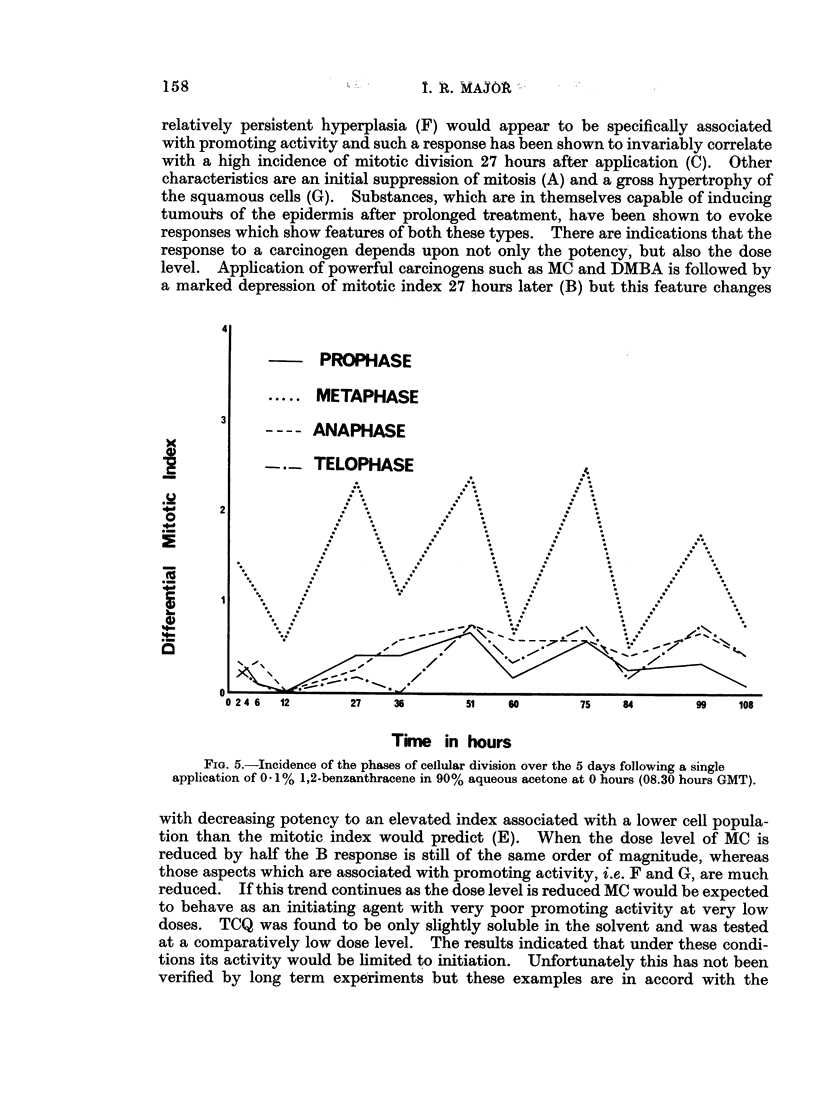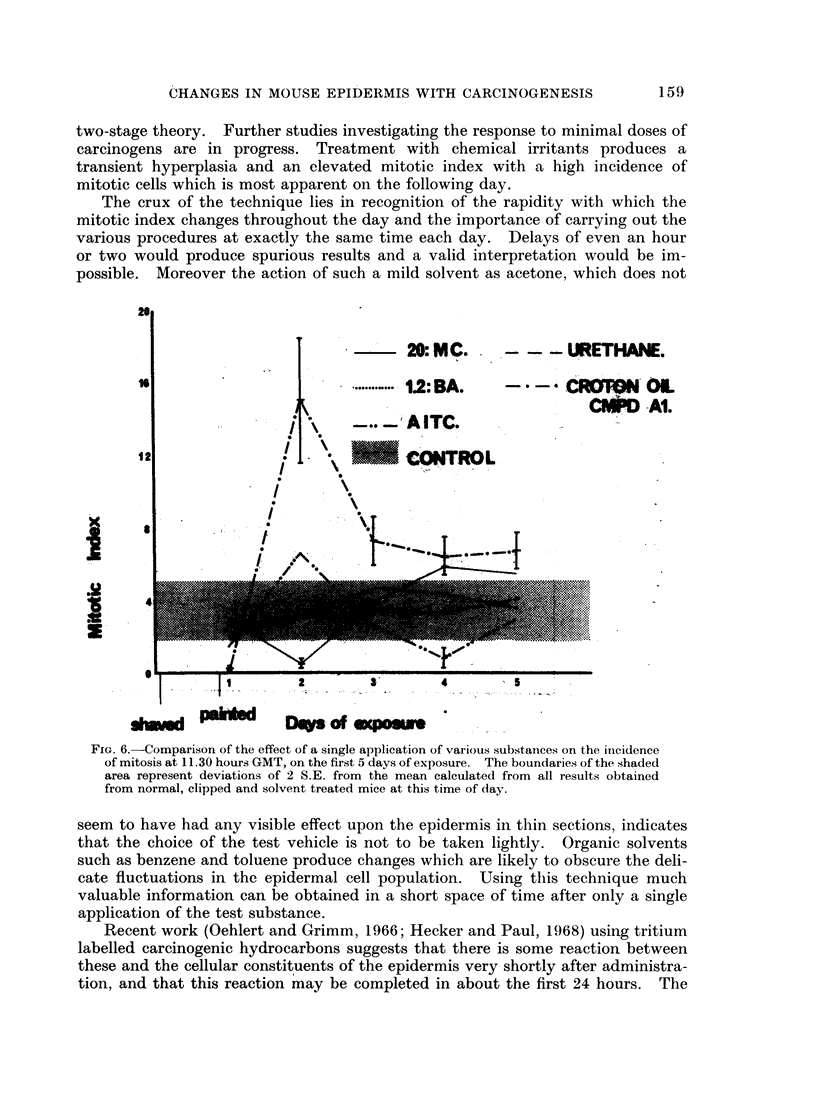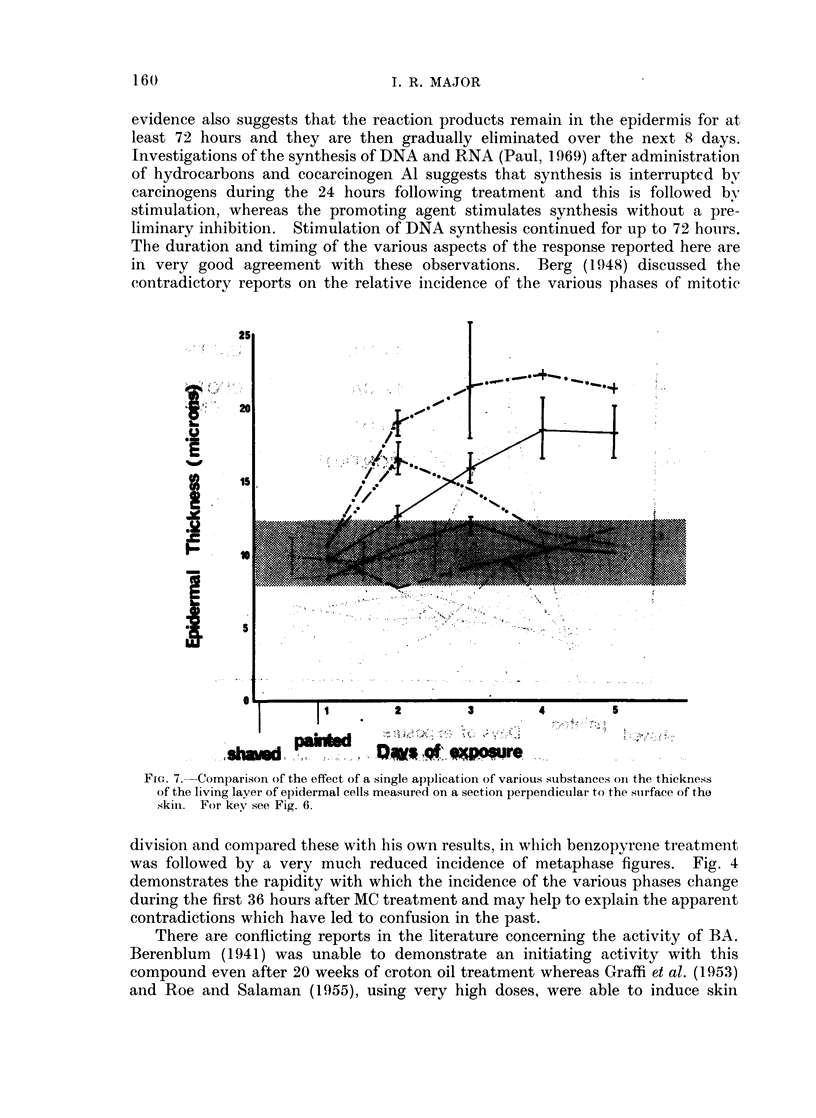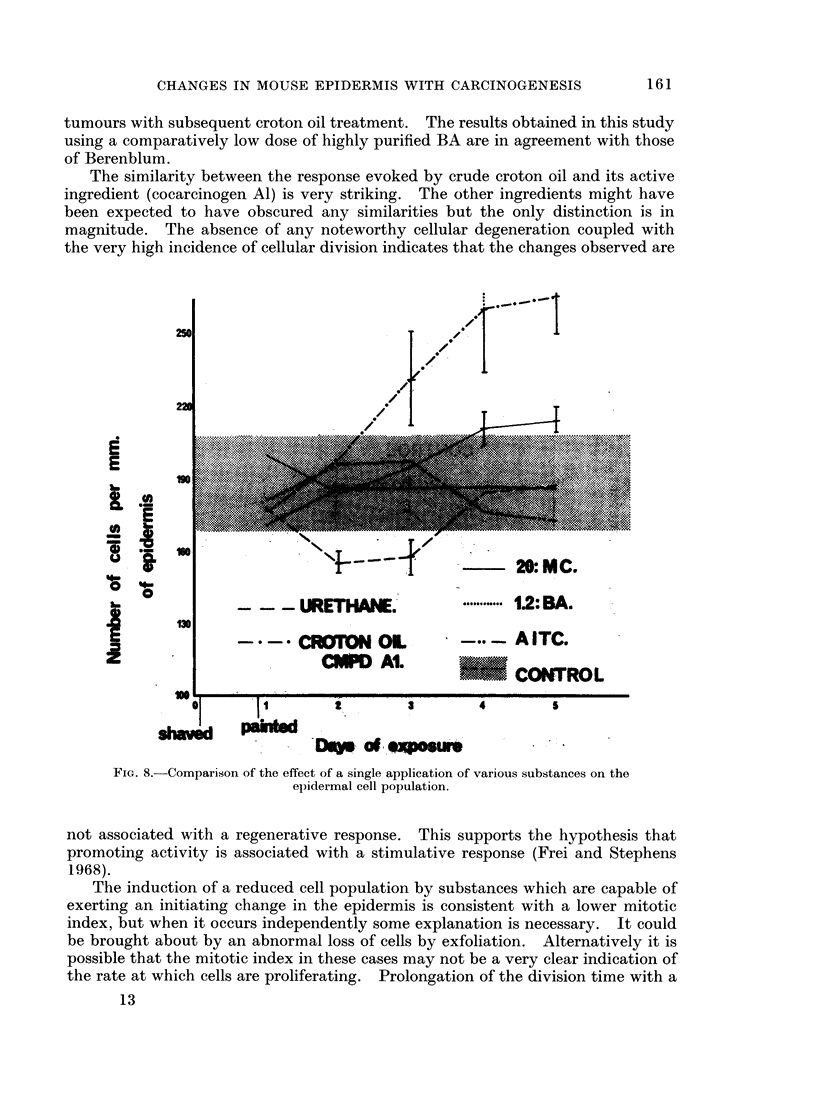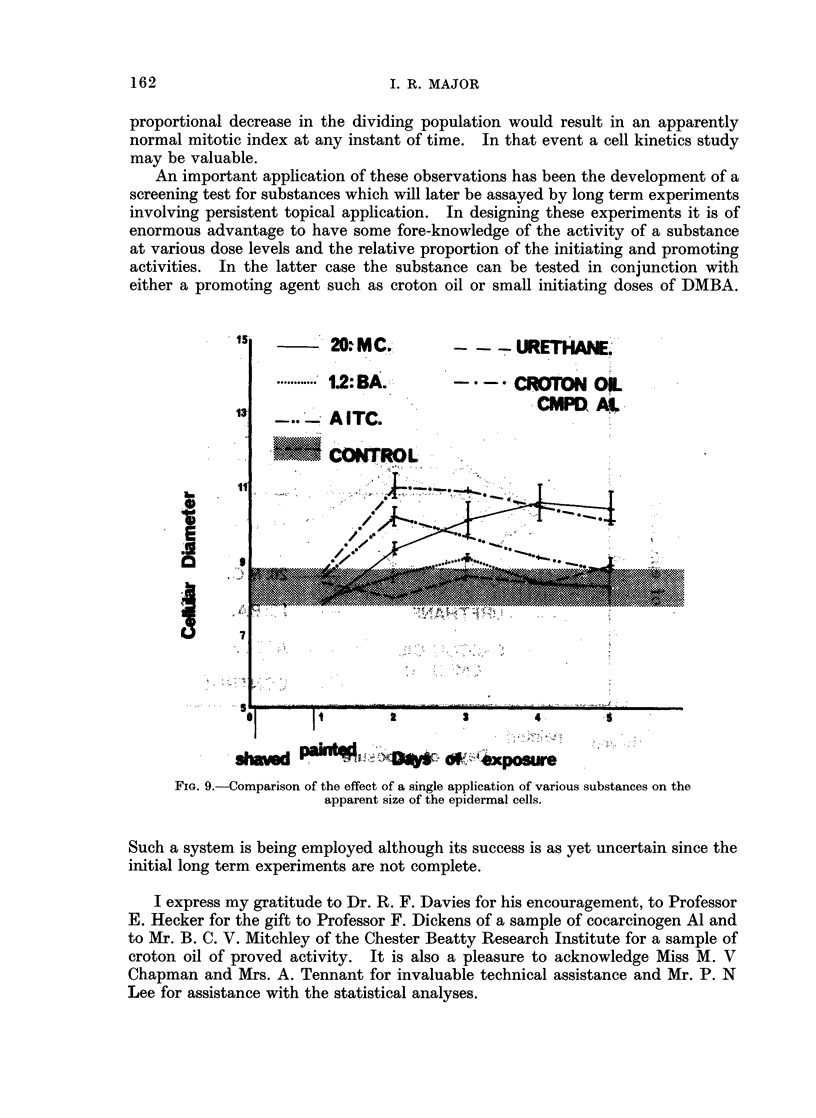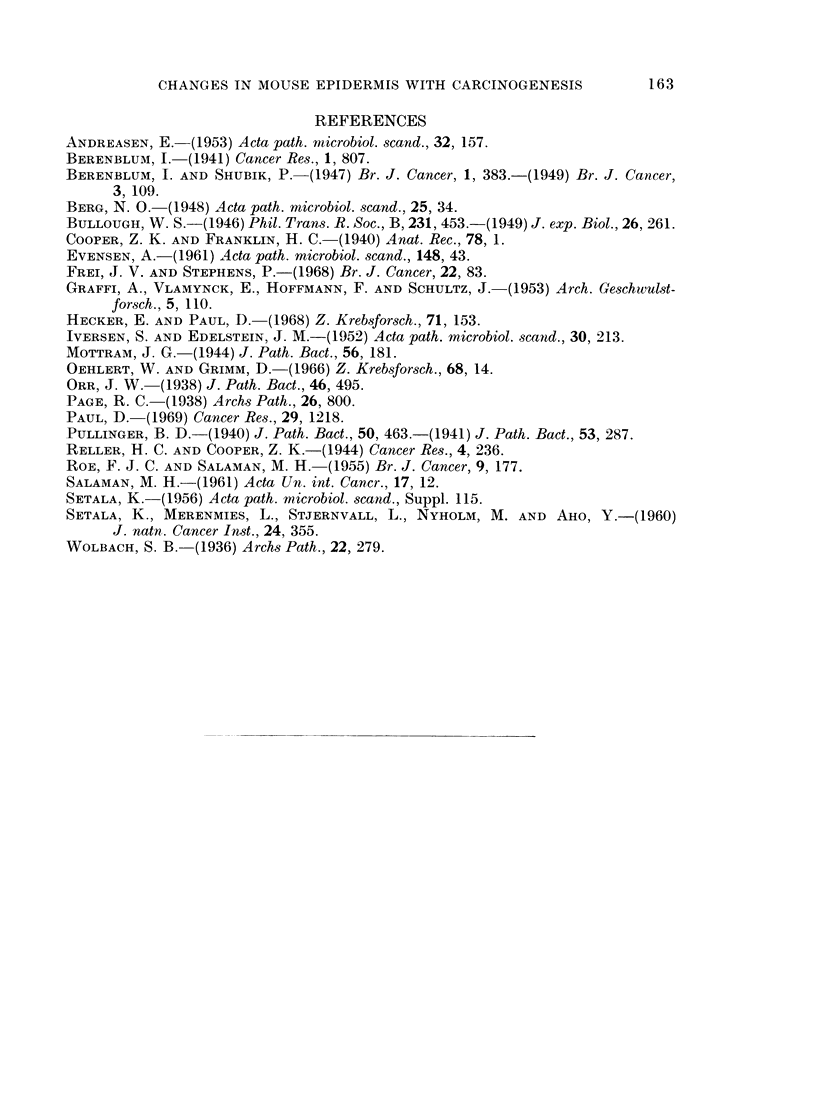# Correlation of Initial Changes in the Mouse Epidermal Cell Population with Two Stage Carcinogenesis—A Quantitative Study

**DOI:** 10.1038/bjc.1970.18

**Published:** 1970-03

**Authors:** I. R. Major

## Abstract

The behaviour of the normal epidermis of mice on the first 5 days of exposure to a single application of carcinogens and cocarcinogens has been investigated by simple quantitative measurements of cell population, size of cells and thickness of the epidermis. Irritant substances and promoting agents both produce cellular hypertrophy but the respective responses can be distinguished by the much greater incidence of degenerate cells associated with irritant treatment. Urethane treatment is characterized by induction of a transient hypoplasia which is not in agreement with the level of cellular division. This response has also been demonstrated after treatment with mild carcinogens or low doses of potent carcinogens. Higher dose levels are followed by a reduction in the mitotic index after about 27 hours. The possibility of developing a preliminary screening test for carcinogenic substances is discussed in the light of these observations.


### CORRELATION OF INITIAL CHANGES IN THE MOUSE EPIDERMAL CELL POPULATION WITH TWO STAGE CARCINOGENESIS—A QUANTITATIVE STUDY

I. R. MAJOR

*From the Tobacco Research Council Laboratories, Harrogate*

Received for publication December 19, 1969

**SUMMARY.—The behaviour of the normal epidermis of mice on the first 5 days of exposure to a single application of carcinogens and cocarcinogens has been investigated by simple quantitative measurements of cell population, size of cells and thickness of the epidermis. Irritant substances and promoting agents both produce cellular hypertrophy but the respective responses can be distinguished by the massive and persistent hyperplasia associated with promoting activity and the much greater incidence of degenerate cells associated with irritant treatment. Urethane treatment is characterized by induction of a transient hypoplasia which is not in agreement with the level of cellular division. This response has also been demonstrated after treatment with mild carcinogens or low doses of potent carcinogens. Higher dose levels are followed by a reduction in the mitotic index after about 27 hours. The possibility of developing a preliminary screening test for carcinogenic substances is discussed in the light of these observations.**

THE application of a carcinogen to the skin of mice induces a thickening of the epidermis within a few hours, but this is not a specific response since it is commonly found after physical or chemical damage. Wolbach (1936), Orr (1938) and Page (1938) were unable to distinguish the response induced by carcinogens from that induced by non-carcinogenic irritants and considered it to be part of a regenerative process. Pullinger (1940, 1941) described the changes which occurred each day in the epidermal cell population drawing attention to the increase in volume of nuclei and cells, the disturbance in the incidence of mitotic division and the discrepancy between the comparatively small proportion of degenerative cells and the subsequent strong hyperplasia. She believed that these and other characteristics could be used to distinguish a carcinogen from a non-carcinogen. Several workers (Reller and Cooper, 1944; Iversen and Edelstein, 1952; Evensen, 1961) also noted a transitory reduction in mitotic index followed by an increase at a later stage. Other workers failed to recognize this depression until it was realized that mitotic index in the mouse epidermis is associated with a pronounced diurnal rhythm (Cooper and Franklin, 1940). Even when this problem had been resolved it was shown that many carcinogens do not reduce the mitotic index shortly after exposure.

Mottram (1944) and Berenblum and Shubik (1947, 1949) proposed a two-stage mechanism for carcinogenesis and it is now generally accepted that initiation brings about some form of cellular change which remains latent until it is subsequently forced to appear as a tumour by persistent exposure to a promoting agent.

Pure initiating agents are known and these when applied to the skin appear to have no visible effect upon the epidermis (Roe and Salaman, 1955), but the promoting stage is thought to be characterized by induction of a marked and sustained hyperplasia brought about by stimulation of cell proliferation and distinct from a regenerative hyperplasia (Setala, 1956; Salaman, 1961; Frei and Stephens, 1968).

The purpose of this investigation was to examine the progress of the response after treatment with carcinogens, cocarcinogens and irritant substances using quantitative techniques in order to be as objective as possible. The method of investigation was confined to simple measurements of cell population, size of cells and epidermal thickness at selected times after treatment in the hope that this would reveal characteristics of the response which were specific. There is now reason to believe that initiating agents do evoke a characteristic response and that this can readily be distinguished from the response produced by a promoting agent. A distinction between the response to a non-carcinogenic irritant and the response to a promoting agent has also been demonstrated but because the evidence is based on only a few experiments it is not certain whether this distinction can yet be regarded as applying generally.

### MATERIALS AND METHODS

*Animals*

The mice were obtained from Imperial Chemical Industries Ltd., and only 3 month old male mice were used because mitotic index is thought to vary with age (Bullough, 1949), the hair cycle is believed to be in the resting phase at this age (Andreasen, 1953) and mitotic index is believed to be associated with the oestrous cycle in female mice (Bullough, 1946). They were isolated in an air-conditioned room at a constant temperature (20–21° C.) and provided with Oxoid Breeding Diet pellets and water *ad libitum*. Each mouse was housed in a separate galvanized iron box on sterile sawdust.

*Chemicals*

These were obtained from the following sources: Professor E. Hecker, Biochemisches Institut, Heidelberg (cocarcinogen Al); Chester Beatty Research Institute (croton oil); University of Nottingham (tricycloquinazoline (TCQ)); British Drug Houses Ltd. (urethane (ethyl carbamate), 1,2-benzanthracene (BA)); Fluka A.G., Buchs, Switzerland (20-methylcholanthrene (MC) 1,2,5,6-dibenzanthracene (DBA)); Koch Light Laboratories (9,10-dimethyl 1,2-benzanthracene (DMBA); 1,2,3,4-DBA; acridine); Middletons Ltd., Stockton on Tees (allyl isothiocyanate (AITC)); B. Newton Maine Ltd. (1,2,7,8-DBA).

The BA was purified by repeated recrystallization from ethanol. The other agents were used without additional purification.

*Methods of measurement*

*Epidermal thickness.*—The width of the epidermis was measured with a scale on a graticule incorporated in the microscope eyepiece. The graticule divisions represented 1·042 μm. at the magnification used. During processing of the skins there is a tendency for the stratum corneum to become displaced from the surface in some areas so only the thickness of the non-cornified layers was measured in this way.

*Number of cells per millimetre of epidermis.*—The graticule is also inscribed with a square around the measuring scale and the number of nuclei within this area was enumerated at 10 different locations on each section of epidermis. These locations correspond to readings on the vernier scale of the microscope stage and they were preselected to avoid subjective errors. Only nuclei in the interfollicular epidermis were counted.

*Mitotic index.*—Ten groups of 100 nucleated cells were differentiated at 1 mm. intervals down the epidermis of 2 sections from each mouse.

*Cellular diameter.*—The size of cells was calculated from the epidermal thickness and the number of cells per millimetre of epidermis using the following formula:

$$\text{Diameter} = 2 \times \sqrt{\frac{\text{Mean thickness} \times 1000}{\text{No. of cells per mm.} \times \pi}}$$

This is a very loose approximation since the cells are assumed to be perfectly spherical and the intercellular space is ignored. If this parameter is regarded as an indication of changes in cell size and not examined too critically, the first assumption can be accepted. Setala *et al.* (1960), using the electron microscope, showed that less than one fifth of the greatest increase in epidermal thickness, observed after treatment with Tween 60, is due to increase in intercellular space. However since this calculated value is directly associated with two of the measurements it should be interpreted with caution.

*Experimental procedure*

About 24 hours before treatment the hair from a strip of skin about $1\frac{1}{2}$ cm. wide along the dorsal midline of the mice from the nape of the neck to the base of the tail was removed by electric clippers. Great care was taken to avoid damaging the skin and on the rare occasions when this did happen the mouse was discarded. The clipping was always started at 10.00 hours GMT to avoid changes which might be associated with the diurnal rhythm. Painting was carried out at 08.30 hours on the following day when a volume of 0·3 ml. of the test solution was delivered directly from the syringe of a Jencons repette to form a shallow lake bound by the unclipped hair on the edge of the treated area and the solvent was allowed to evaporate. In all experiments the test substances were dissolved in either a mixture of acetone and distilled water (9 : 1 by volume) or a mixture of acetone and isopropyl alcohol (4 : 1 by volume). At specific times, which were rigidly adhered to in all cases, the mice were killed and a rectangular area of skin, with the longer side at right angles to the dorsal line of the animal and slightly wider than the treated area, was removed from the lower thoracic region of each mouse. The under surface of the skin was applied to thin card to minimize distortion during fixation in Zenker's fluid containing 5% acetic acid. This work is confined to an examination of the response to a single application of each test substance.

Seventy-two mice were painted with the aqueous acetone solvent and they were then sampled in groups of 6 at predetermined intervals of time. Four groups were killed on the day of painting and thereafter sampling was carried out twice a day at 11.30 hours and 20.30 hours. A group of 24 mice were handled, one at a time, at the same times as the solvent control animals were clipped and painted.

TABLE I.—*Effect of Clipping and Application of Solvent on the Four Parameters Compared with Values Obtained from Normal Untreated Animals. Mean Values are Followed by 68% Fiducial Limits in Brackets*

| | Hours after painting (in Series c) | | | |
|---|---|---|---|---|
| | 3 | 12 | 27 | 75 |
| Epidermal thickness (μm.) | | | | |
| (a) Normal untreated | 8·5 (7·8–9·3) | 7·6 (7·4–7·8) | 8·4 (7·6–9·2) | 9·5 (9·0–9·9) |
| (b) Clipped untreated | 9·3 (8·8–9·9) | 8·1 (7·6–8·7) | 10·5 (9·6–11·5) | 8·7 (8·1–9·3) |
| (c) Clipped and solvent treatment | 9·8 (8·9–10·7) | 10·4 (10·0–10·9) | 9·0 (8·5–9·6) | 11·4 (10·9–11·9) |
| Mitotic index | | | | |
| (a) Normal untreated | 0·32 (0·27–0·37) | 0·05 (0·03–0·07) | 0·29 (0·26–0·32) | 0·20 (0·18–0·22) |
| (b) Clipped untreated | 0·36 (0·29–0·43) | 0·18 (0·14–0·22) | 0·38 (0·31–0·45) | 0·38 (0·34–0·42) |
| (c) Clipped and solvent treatment | 0·38 (0·28–0·48) | 0·11 (0·08–0·14) | 0·48 (0·38–0·58) | 0·37 (0·31–0·43) |
| No. of cells per millimetre | | | | |
| (a) Normal untreated | 183 (177–189) | 171 (160–183) | 177 (168–187) | 194 (187–202) |
| (b) Clipped untreated | 184 (175–192) | 181 (172–192) | 189 (180–199) | 179 (174–185) |
| (c) Clipped and solvent treatment | 197 (187–208) | 195 (186–205) | 173 (166–180) | 199 (195–203) |
| Apparent cellular diameter (μm.) | | | | |
| (a) Normal untreated | 7·7 (7·4–8·0) | 7·5 (7·1–8·0) | 7·8 (7·4–8·1) | 7·9 (7·8–8·0) |
| (b) Clipped untreated | 8·0 (7·9–8·1) | 7·6 (7·4–7·7) | 8·4 (8·1–8·7) | 7·8 (7·6–8·0) |
| (c) Clipped and solvent treatment | 7·9 (7·7–8·2) | 8·2 (8·1–8·4) | 8·1 (8·0–8·3) | 8·5 (8·3–8·8) |

They were then divided into 4 groups and these were sampled at the times equivalent to those sampled 4, 12, 27 and 75 hours after solvent treatment. Another group of 24 mice were treated in exactly the same way but in this case the dorsal midline of each mouse was clipped in the same way and at the same time of day as the solvent controls. The schedule used in these experiments formed the basic design for further experimentation but, as the general trends became apparent, slight modifications were made These are described at appropriate points in the next section.

FIG. 1.—Changes in the mitotic index throughout a 24 hour period.

RESULTS

The results from the 3 control experiments are compared in Table I. The mitotic counts showed remarkably plainly the way in which cellular division is associated with diurnal variation. The evening count carried out on the solvent controls on each day was consistently lower than that at midday, resulting in 5 maximum points. On the first day and the last 2 days of sampling the results were the same but the maximum on the second day was somewhat higher, and that on the third day somewhat lower than the average. It was not clear whether this was associated with the solvent treatment. The other parameters were all fairly constant and did not appear to be associated with a diurnal rhythm.

The measurements of epidermal thickness and the mitotic counts were compared by analyses of variance. These confirmed that the thickness of the epidermis did not vary significantly over the experimental period, but clipping and subsequent application of the solvent brought about an increase in thickness. There was a slight, but insignificant, increase after clipping but a significant increase

($P < 0{\cdot}01$) after both clipping and solvent treatment. In association with this it was shown that clipping and painting with the solvent produced a significantly higher level ($P < 0{\cdot}01$) of cellular division. The changes in mitotic index associated with the diurnal rhythm were also shown to be significant ($P < 0{\cdot}01$).

The mean mitotic index in the epidermis was determined in 12 groups of 6 mice which had been killed at 2-hourly intervals throughout a complete 24-hour period. These results are presented in Fig. 1. It will be seen that the mitotic

FIG. 2.—Effect of a single application of various test substances on the epidermal mitotic index over the next 5 days. Painting was carried out at 08.30 hours GMT (0 hours on the abscissa). The control mice were painted with 90% aqueous actone and the curve follows a diurnal rhythm.

index followed a precise diurnal rhythm reaching a maximum at about 14.30 hours and a minimum in the early hours of the morning. Thus the importance of sampling at exactly the same time of day was confirmed.

A non-carcinogenic irritant (5% AITC), a carcinogenic polycyclic aromatic hydrocarbon (0·1% MC) and a non-carcinogenic hydrocarbon (0·1% BA) were tested using exactly the same experimental procedure as that used in the solvent control experiment. The results of the mitotic counts are compared in Fig. 2. The mitotic figures were also differentiated into the various phases and these results are shown in Fig. 3, 4 and 5. There is no evidence to suggest that treatment with the polycyclic hydrocarbons has influenced the duration of any of the phases of mitotic division.

The response of the epidermis to the initiating agent, urethane (20%) and the promoting agent, cocarcinogen Al (0·00125%) was determined. In these 2 cases sampling was carried out once each day at 11.30 hours. All the results obtained at this time of day are compared in Fig. 6–9.

At this stage it was apparent that the important differences between the respective responses occurred on the first, second and fourth days of exposure. Moreover the characteristic features could still be plainly recognized in a sample of only 4 mice. To determine whether these changes were really characteristic of whole groups, and not just the single examples which had already been examined, several more substances were tested and it became apparent that definite response

FIG. 3.—Incidence of the phases of cellular division over the 5 days following a single application of 90% aqueous acetone at 0 hours (08.30 hours GMT).

patterns were emerging. Certain aspects of these appeared to be of particular relevance and importance and these were coded as follows:

A. A reduction in mitotic index 3 hours after painting.
B. An even greater reduction in mitotic index 27 hours after painting.
C. An elevated mitotic index 27 hours after painting.
D. Epidermal hypoplasia 27 hours after painting.
E. A relatively low epidermal cell population which is not in agreement with a high mitotic index 27 hours after painting.
F. Epidermal hyperplasia 75 hours after painting.
G. Hypertrophy of the cells at any time after application.

H. A high incidence of degenerate cells 27 hours after painting.
J. A marked decrease in the cell population between 27 hours and 75 hours after painting.

The epidermal response to all the substances which had been tested is presented in Table II on the basis of this coding system. All other results obtained at these times (*i.e.* 3, 27 and 75 hours after painting) were either within the normal control range or were of no relevance to the interpretation of the response patterns.

FIG. 4.—Incidence of the phases of cellular division over the 5 days following a single application of 0·1% 20-methylcholanthrene in 90% aqueous acteone at 0 hours (08.30 hours GMT). Since none of the phases occurs in unusually high proportion at any time it would appear that the carcinogen does not interfere with the actual mechanism of mitosis.

### DISCUSSION

The response patterns which have emerged demonstrate particular features which can be recognized as being specifically, characteristically or occasionally associated with the types of activity under investigation. It seems likely that a reduction in the epidermal cell population (D and E), whether it is associated with a reduced mitotic index (A and B) or is brought about by some unknown mechanism, is a specific response to treatment with an initiating agent. The response to topical application of urethane is apparent between 27 hours and 51 hours after application, whereas the response associated with promoting activity proceeds beyond this time and is still apparent at least 75 hours after treatment A

TABLE II.—*Changes in the Epidermis at* 11.30 *Hours GMT on the* 1*st*, 2*nd and* 4*th Days Following a Single Application of Various Test Substances at* 08.30 *Hours on Day* 1

| Test substance | Dose level (%) | Solvent | Group size | Hours after painting: 3 | 27 | | | | 75 | | Response pattern (see text) |
|---|---|---|---|---|---|---|---|---|---|---|---|
| | | | | M | M | N | S | D | N | S | |
| 20-Methylcholanthrene | 0·1 | acetone/water | 6 | 2·92 | 0·42 | 184 | 9·3 | — | 211 | 10·6 | A B F G |
| 20-Methylcholanthrene | 0·05 | acetone/water | 4 | 4·38 | 0·50 | 165 | 9·2 | 2·25 | 176 | 10·4 | B D G |
| 9,10-Dimethylbenzanthracene | 0·1 | acetone/water | 4 | 4·50 | 0·75 | 177 | 8·8 | 3·13 | 167 | 12·1 | B G |
| 1,2,5,6-Dibenzanthracene | 0·1 | acetone/IPA | 4 | 3·25 | 2·88 | 164 | 8·9 | 1·00 | 164 | 9·2 | B D G |
| 1,2,7,8-Dibenzanthracene | 0·1 | acetone/water | 4 | 2·50 | 6·88 | 180 | 8·5 | 0·63 | 170 | 9·5 | A C E G |
| 1,2,3,4-Dibenzanthracene | 0·1 | acetone/water | 4 | 3·25 | 4·25 | 157 | 8·6 | 2·50 | 168 | 9·0 | C D E |
| Tricycloquinazoline | 0·0086 | acetone/IPA | 4 | 4·38 | 5·50 | 153 | 8·9 | 0·63 | 176 | 8·7 | C D E |
| Urethane | 20·0 | acetone/water | 6 | 2·58 | 3·00 | 155 | 8·0 | 0·50 | 184 | 8·4 | A D |
| Cocarcinogen Al. | 0·00125 | acetone/water | 6 | 0·17 | 15·00 | 198 | 11·0 | 0·17 | 260 | 10·5 | A C F G |
| Croton oil | 0·1 | acetone/water | 4 | 0·75 | 10·88 | 208 | 10·2 | 0·88 | 216 | 9·4 | A C F G |
| 1,2-Benzanthracene | 0·1 | acetone/water | 6 | 1·93 | 3·17 | 186 | 8·6 | — | 187 | 8·4 | A |
| Acridine | 0·1 | acetone/water | 4 | 2·38 | 2·75 | 192 | 8·4 | 2·00 | 158 | 8·3 | A J |
| Allyl isothiocyanate | 5·0 | acetone/water | 6 | 0·75 | 6·58 | 196 | 10·3 | 7·83 | 176 | 9·1 | A C G H J |

M—mean number of mitotic figures per 1000 nucleated cells.

N—mean number of nucleated cells per millimetre of epidermis.

S—size of cells expressed as apparent cellular diameter.

D—mean number of pyknotic nuclei per 1000 nucleated cells.

IPA—isopropyl alcohol.

relatively persistent hyperplasia (F) would appear to be specifically associated with promoting activity and such a response has been shown to invariably correlate with a high incidence of mitotic division 27 hours after application (C). Other characteristics are an initial suppression of mitosis (A) and a gross hypertrophy of the squamous cells (G). Substances, which are in themselves capable of inducing tumours of the epidermis after prolonged treatment, have been shown to evoke responses which show features of both these types. There are indications that the response to a carcinogen depends upon not only the potency, but also the dose level. Application of powerful carcinogens such as MC and DMBA is followed by a marked depression of mitotic index 27 hours later (B) but this feature changes

FIG. 5.—Incidence of the phases of cellular division over the 5 days following a single application of 0·1% 1,2-benzanthracene in 90% aqueous acetone at 0 hours (08.30 hours GMT).

with decreasing potency to an elevated index associated with a lower cell population than the mitotic index would predict (E). When the dose level of MC is reduced by half the B response is still of the same order of magnitude, whereas those aspects which are associated with promoting activity, *i.e.* F and G, are much reduced. If this trend continues as the dose level is reduced MC would be expected to behave as an initiating agent with very poor promoting activity at very low doses. TCQ was found to be only slightly soluble in the solvent and was tested at a comparatively low dose level. The results indicated that under these conditions its activity would be limited to initiation. Unfortunately this has not been verified by long term experiments but these examples are in accord with the two-stage theory. Further studies investigating the response to minimal doses of carcinogens are in progress. Treatment with chemical irritants produces a transient hyperplasia and an elevated mitotic index with a high incidence of mitotic cells which is most apparent on the following day.

The crux of the technique lies in recognition of the rapidity with which the mitotic index changes throughout the day and the importance of carrying out the various procedures at exactly the same time each day. Delays of even an hour or two would produce spurious results and a valid interpretation would be impossible. Moreover the action of such a mild solvent as acetone, which does not

FIG. 6.—Comparison of the effect of a single application of various substances on the incidence of mitosis at 11.30 hours GMT, on the first 5 days of exposure. The boundaries of the shaded area represent deviations of 2 S.E. from the mean calculated from all results obtained from normal, clipped and solvent treated mice at this time of day.

seem to have had any visible effect upon the epidermis in thin sections, indicates that the choice of the test vehicle is not to be taken lightly. Organic solvents such as benzene and toluene produce changes which are likely to obscure the delicate fluctuations in the epidermal cell population. Using this technique much valuable information can be obtained in a short space of time after only a single application of the test substance.

Recent work (Oehlert and Grimm, 1966; Hecker and Paul, 1968) using tritium labelled carcinogenic hydrocarbons suggests that there is some reaction between these and the cellular constituents of the epidermis very shortly after administration, and that this reaction may be completed in about the first 24 hours. The evidence also suggests that the reaction products remain in the epidermis for at least 72 hours and they are then gradually eliminated over the next 8 days. Investigations of the synthesis of DNA and RNA (Paul, 1969) after administration of hydrocarbons and cocarcinogen Al suggests that synthesis is interrupted by carcinogens during the 24 hours following treatment and this is followed by stimulation, whereas the promoting agent stimulates synthesis without a preliminary inhibition. Stimulation of DNA synthesis continued for up to 72 hours. The duration and timing of the various aspects of the response reported here are in very good agreement with these observations. Berg (1948) discussed the contradictory reports on the relative incidence of the various phases of mitotic

FIG. 7.—Comparison of the effect of a single application of various substances on the thickness of the living layer of epidermal cells measured on a section perpendicular to the surface of the skin. For key see Fig. 6.

division and compared these with his own results, in which benzopyrene treatment was followed by a very much reduced incidence of metaphase figures. Fig. 4 demonstrates the rapidity with which the incidence of the various phases change during the first 36 hours after MC treatment and may help to explain the apparent contradictions which have led to confusion in the past.

There are conflicting reports in the literature concerning the activity of BA. Berenblum (1941) was unable to demonstrate an initiating activity with this compound even after 20 weeks of croton oil treatment whereas Graffi *et al.* (1953) and Roe and Salaman (1955), using very high doses, were able to induce skin tumours with subsequent croton oil treatment. The results obtained in this study using a comparatively low dose of highly purified BA are in agreement with those of Berenblum.

The similarity between the response evoked by crude croton oil and its active ingredient (cocarcinogen Al) is very striking. The other ingredients might have been expected to have obscured any similarities but the only distinction is in magnitude. The absence of any noteworthy cellular degeneration coupled with the very high incidence of cellular division indicates that the changes observed are

FIG. 8.—Comparison of the effect of a single application of various substances on the epidermal cell population.

not associated with a regenerative response. This supports the hypothesis that promoting activity is associated with a stimulative response (Frei and Stephens 1968).

The induction of a reduced cell population by substances which are capable of exerting an initiating change in the epidermis is consistent with a lower mitotic index, but when it occurs independently some explanation is necessary. It could be brought about by an abnormal loss of cells by exfoliation. Alternatively it is possible that the mitotic index in these cases may not be a very clear indication of the rate at which cells are proliferating. Prolongation of the division time with a proportional decrease in the dividing population would result in an apparently normal mitotic index at any instant of time. In that event a cell kinetics study may be valuable.

An important application of these observations has been the development of a screening test for substances which will later be assayed by long term experiments involving persistent topical application. In designing these experiments it is of enormous advantage to have some fore-knowledge of the activity of a substance at various dose levels and the relative proportion of the initiating and promoting activities. In the latter case the substance can be tested in conjunction with either a promoting agent such as croton oil or small initiating doses of DMBA.

FIG. 9.—Comparison of the effect of a single application of various substances on the apparent size of the epidermal cells.

Such a system is being employed although its success is as yet uncertain since the initial long term experiments are not complete.

I express my gratitude to Dr. R. F. Davies for his encouragement, to Professor E. Hecker for the gift to Professor F. Dickens of a sample of cocarcinogen Al and to Mr. B. C. V. Mitchley of the Chester Beatty Research Institute for a sample of croton oil of proved activity. It is also a pleasure to acknowledge Miss M. V Chapman and Mrs. A. Tennant for invaluable technical assistance and Mr. P. N Lee for assistance with the statistical analyses.

### REFERENCES

Andreasen, E.—(1953) *Acta path. microbiol. scand.*, **32**, 157.
Berenblum, I.—(1941) *Cancer Res.*, **1**, 807.
Berenblum, I. and Shubik, P.—(1947) *Br. J. Cancer*, **1**, 383.—(1949) *Br. J. Cancer*, **3**, 109.
Berg, N. O.—(1948) *Acta path. microbiol. scand.*, **25**, 34.
Bullough, W. S.—(1946) *Phil. Trans. R. Soc.*, B, **231**, 453.—(1949) *J. exp. Biol.*, **26**, 261.
Cooper, Z. K. and Franklin, H. C.—(1940) *Anat. Rec.*, **78**, 1.
Evensen, A.—(1961) *Acta path. microbiol. scand.*, **148**, 43.
Frei, J. V. and Stephens, P.—(1968) *Br. J. Cancer*, **22**, 83.
Graffi, A., Vlamynck, E., Hoffmann, F. and Schultz, J.—(1953) *Arch. Geschwulstforsch.*, **5**, 110.
Hecker, E. and Paul, D.—(1968) *Z. Krebsforsch.*, **71**, 153.
Iversen, S. and Edelstein, J. M.—(1952) *Acta path. microbiol. scand.*, **30**, 213.
Mottram, J. G.—(1944) *J. Path. Bact.*, **56**, 181.
Oehlert, W. and Grimm, D.—(1966) *Z. Krebsforsch.*, **68**, 14.
Orr, J. W.—(1938) *J. Path. Bact.*, **46**, 495.
Page, R. C.—(1938) *Archs Path.*, **26**, 800.
Paul, D.—(1969) *Cancer Res.*, **29**, 1218.
Pullinger, B. D.—(1940) *J. Path. Bact.*, **50**, 463.—(1941) *J. Path. Bact.*, **53**, 287.
Reller, H. C. and Cooper, Z. K.—(1944) *Cancer Res.*, **4**, 236.
Roe, F. J. C. and Salaman, M. H.—(1955) *Br. J. Cancer*, **9**, 177.
Salaman, M. H.—(1961) *Acta Un. int. Cancr.*, **17**, 12.
Setala, K.—(1956) *Acta path. microbiol. scand.*, Suppl. 115.
Setala, K., Merenmies, L., Stjernvall, L., Nyholm, M. and Aho, Y.—(1960) *J. natn. Cancer Inst.*, **24**, 355.
Wolbach, S. B.—(1936) *Archs Path.*, **22**, 279.